# Different Effects of Annealing on Microstructure Evolution and SERS Performance for Cu–Cr Alloy Film and Bulk Alloy

**DOI:** 10.3390/ma12182990

**Published:** 2019-09-16

**Authors:** Xiaoxue Huang, Haoliang Sun, Jun Shen, Kai Cui, Guangxin Wang

**Affiliations:** 1 School of Materials Science and Engineering, Henan University of Science and Technology, Luoyang 471003, China; hxxhkd@163.com (X.H.); shenjudw@163.com (J.S.); ckdrdyx@163.com (K.C.); 2 Collaborative Innovation Center of Nonferrous Metals Henan Province, Luoyang 471003, China

**Keywords:** Cu–Cr alloy film, Cu–Cr bulk alloy, polyimide, annealing, particle, SERS substrate

## Abstract

Copper–chromium alloy film and Cu–Cr bulk alloy were obtained using magnetron sputtering and vacuum smelting. Experimental results indicated that Cu–Cr bulk alloy and alloy films having different residual stress and atomic diffusion exhibit a significant difference in microstructure evolution behaviors after annealing. Numerous polyhedral Cu particles and dendritic Cr particles precipitated on the surface of annealed Cu–Cr alloy film and as–cast Cu–Cr bulk alloy, respectively. Cu particles were formed under the driving of energy and residual stress in the film. The effect of annealing temperature and Cr content on the size and quantity of Cu particles is discussed. Cr particles precipitated on the bulk alloy due to the low solid solubility of Cr in Cu, and the crystallinity of Cu grains promoted the diffusion of Cr atoms. The surface–enhanced Raman scattering (SERS) intensity of the Cu–14.6%Cr alloy film was obviously higher than that of the Cu–14.2%Cr bulk alloy. The particles/film composite structure possessed the appropriate particle number, surface roughness, and interstitial gap, as opposed to the bulk material, to effectively improve SERS enhancement.

## 1. Introduction

Copper (Cu) and copper alloys have gradually replaced aluminum (Al) as the interconnect materials widely used in various fields due to their excellent electrical conductivity and good electromigration resistance, as the size of integrated circuits decreases [1,2,3]. It is well–known that composites with a nano–grained or nano–dispersed structure have good mechanical properties [4]. Normally, the high–melting–point elements have low solid solubility in Cu, so it is difficult to form high–performance Cu alloys. Particularly, the Cu–Cr system shows a limited solubility range at the equilibrium state, according to the equilibrium–phase diagram [5]. However, Cr is a suitable additional metal to prepare Cu alloys with high strength and great conductivity. Cu–Cr alloys are extensively studied as important materials for railway contact wires and electrodes for spot [6]. Wo et al. [7] have investigated how the precipitation of Cr can strengthen nanocomposite Cr/Cu–Cr multilayer films. Prior work demonstrated that the bulk material and the corresponding thin film material with the same composition might exhibit different microstructure evolution behavior after annealing [8]. The electrical conductivity and tensile strength of a Cu–Cr alloy can be significantly improved after annealing treatment [3]. The Cu–Cr alloy film after annealing will release residual stress and reduce point defects to optimize its mechanical properties [9]. Hence, in this work, different effects of annealing on microstructure evolution and surface–enhanced Raman scattering (SERS) performance of Cu–Cr bulk alloy and alloy film deposited on polyimide were studied comparatively.

## 2. Sample Preparation and Characterization Methods

Cu–Cr alloy films were deposited on polyimide (PI, thickness 125 μm) substrates by magnetron sputtering, using the composite target consisting of a copper (Cu) target (99.99%, purity, Ø50 mm × 4 mm) overlaid with three chromium (Cr) plates (99.99%, purity, 10 mm × 10 mm × 1 mm). Before sputtering, the PI was cleaned several times with acetone and anhydrous ethanol, and pre–sputtering for 20 min was conducted. The base pressure of the vacuum chamber, the sputtering pressure, and power were maintained at 5 × 10^−4^ Pa, 0.4 Pa, and 120 W, respectively. Subsequently, the samples were annealed in a tube furnace with argon. The annealing temperatures were 160 °C and 360 °C for a holding time of 1 h.

Cu–Cr bulk alloys were melted using pure copper (99.99%) and pure chromium (99.96%) as raw materials in a crucible of a vacuum induction melting furnace under high vacuum (10^−3^ Pa). After holding for 30 min, the molten metal was poured into the water–cooled crystallizer to rapidly cool it to the solid phase. Some as–cast Cu–Cr bulk alloys were annealed at 160 °C and 360 °C for 1 hour in a vacuum tubular furnace.

The pure PI and annealed Cu–Cr bulk alloy and Cu–Cr alloy film were immersed in R6G solutions (300 mL) for 30 min and dried naturally by air. A laser micro–Raman spectrometer (Renishaw inVia, Renishaw, London, England) with a laser wavelength of 632.18 nm and a 50× objective (NA = 0.80) was used for analyses. The laser excitation energy, spot, and diffraction grid were 5 mW, 2 μm, and 1200 gr/mm, respectively. The integration time was 1 s.

The surface morphology and phase structure of the Cu–Cr bulk alloy and alloy films were obtained by using a field emission scanning electron microscope (FESEM, JSM–7800F, JEOL Ltd., Tokyo, Japan) and X–ray diffractometer (XRD, D8 advance, Bruker, Karlsruhe, Germany), respectively. Energy–dispersive spectrometer (EDS, JEOL Ltd., Tokyo, Japan) tests were carried out in five areas on the surface of the sample, and the Cu–Cr alloy composition was obtained through the average value of five results.

## 3. Results and Discussion

### 3.1. Structural Properties of Cu–Cr Bulk Alloy and Alloy Films

**Figure 1:**
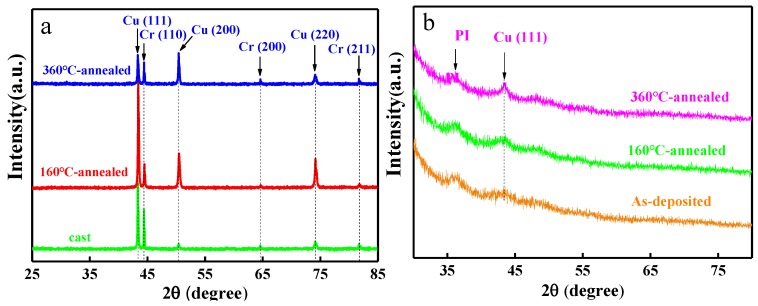
XRD patterns of the Cu–Cr bulk alloy and alloy films: (**a**) Cu–14.2%Cr bulk alloy and (**b**) Cu–14.6%Cr alloy film.

It is difficult to obtain metastable Cu–Cr compounds in Cu–Cr alloys with a low content of Cr at low temperatures [10]. XRD patterns of the Cu–Cr alloy film and bulk alloy are presented in Figure 1. The any compounds peak is not obtained. Figure 1a shows the XRD pattern of as–cast and annealed Cu–14.2%Cr bulk alloy at different annealing temperatures. The crystal diffraction peaks of Cu (111), Cu (200), Cu (220), Cr (110), Cr (200), and Cr (211) were existed as–cast Cu–Cr bulk alloy, indicating the randomly oriented grains. It also is elucidated that the Cr particle precipitated on the surface of the Cu–Cr bulk alloy. Therein, the Cu (111) peak is the preferred orientation. This is because the crystal structure of Cu is face–centered cubic, and (111) as a dense–packed plane, it has the lowest free energy. After 160 °C annealing, the intensities of Cu (111), Cu (200), and Cu (220) diffraction peaks are obviously increased, while that of Cr (110) is decreased, and the other Cr peaks remain unchanged, which demonstrates that the crystallinity of Cu grains improves and promotes the diffusion of Cr atoms. However, the diffraction peak intensity of Cu decreases gradually, and that of Cr increases a little as the annealing temperature is increased to 360 °C, which indicate the further growth of the Cr particles and the diffusion of Cu atoms.

The XRD pattern characteristic of Cu–14.6%Cr alloy film is quite different from that of Cu–14.2%Cr bulk alloy, as shown in Figure 1b. Apart from the obvious substrate (PI ) peaks, the deposited Cu–Cr alloy film possesses peaks in diffuse scattering and a low crystallinity Cu (111) diffraction peak without any Cr peaks, showing near–amorphous thin film. The alloy films (Cu(Zr)–M, M = Zr, Cr, Ni, Co) prepared by magnetron sputtering may be amorphous films with the short– and medium–range order microstructure [11,12]. In the initial stage of alloy film formation, the tendency to form amorphous thin film is higher than that of crystalline, which is mainly affected by activation energy and free energy during the nucleation process. It was found that the peak width of Cu (111) gradually becomes narrower with increasing annealing temperatures, revealing that the crystallinity of the Cu atom increases.

### 3.2. Surface Morphology of Cu–Cr Alloy Films

**Figure 2:**
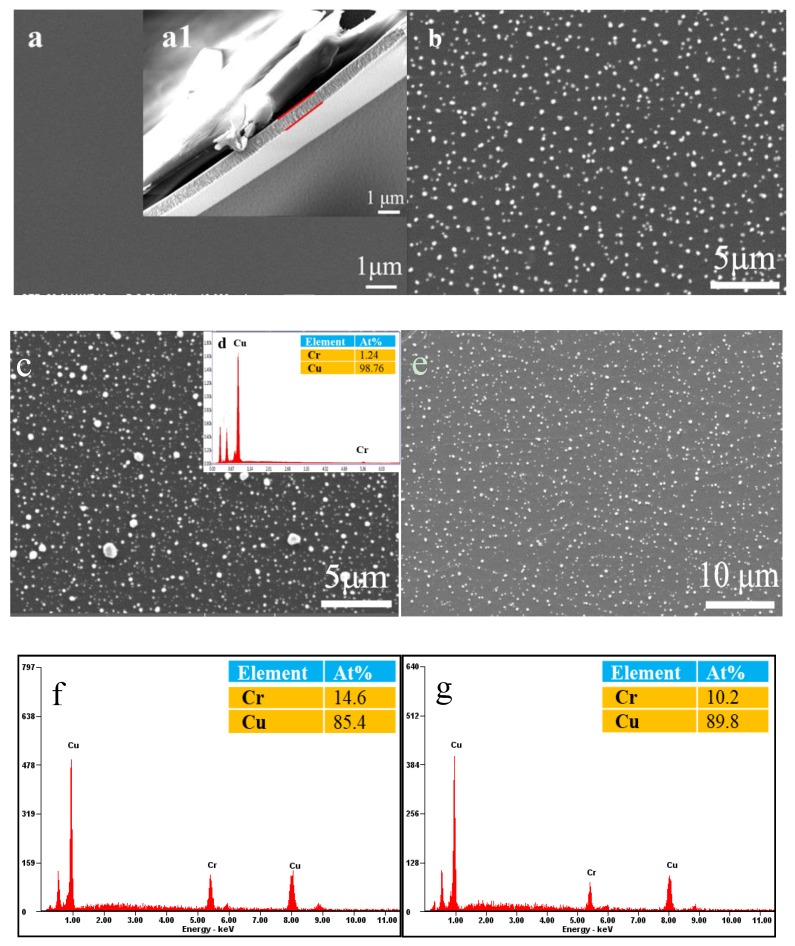
SEM images of as–deposited and annealed Cu–14.6%Cr alloy film with different annealing temperatures: (**a**) as–deposited, (**b**) 160 °C, (**c**) 360 °C, (**a1**) cross–sectional FE–SEM image of deposited Cu–Cr alloy film, (**d**) energy–dispersive spectrometry (EDS) of the particles, (**e**) 160 °C annealed Cu–10.2%Cr alloy film, (**f**) EDS of the Cu–Cr alloy films, and (**g**) EDS of the Cu–Cr alloy films.

Figure 2 shows the surface morphologies of as–deposited and annealed Cu–14.6%Cr alloy films at different temperatures. From the cross–sectional FE–SEM image of deposited Cu–Cr alloy film in Figure 2a1, the film is composed of columnar crystals that are without any defects, have a clear interface, and are uniform in film thickness. The film thickness can reach about 594 nm (red parallel line). The surface of as–deposited Cu–14.6%Cr alloy film is smooth and without any particles, as shown in Figure 2a. However, after annealing, a large number of polyhedral particles (~200 nm) self–formed on the film surface, which is different from Cu–Cr thin film on silicon substrate [13] but the same as the Ag particles self–formed on the Ag–Zr alloy film’s surface in our previous work [14], mainly due to the release of thermal stress existing in the film [15]. The EDS data in Figure 2d indicates that the compositions of these particles are pure Cu elements, excluding the effect of PI substrates. Additionally, for the annealed Cu–14.6%Cr alloy films with the same Cr content and thickness, some Cu particles’ aggregation, growth, size, and number of Cu particles increased monotonously with the increasing annealing temperatures. This tendency is in agreement with the results of XRD in Figure 1b. This can be explained by taking into account the fact that the higher annealing temperature is easier to activate atom diffusion along grain boundaries and surface driven by the relaxation of high tensile stress [16,17]. Furthermore, compared with Cu–Cr alloy film of different Cr content in Figure 2b,e, it was found that the number of Cu particles increases and the size of Cu particles decreases as the Cr content increases. This can be attributed to the fact that larger Cr contents are able to suppress Cu atoms’ diffusion along grain boundary and surface, thus leading to smaller Cu particles [13].

### 3.3. Surface Morphology of Cu–Cr Bulk Alloy

**Figure 3:**
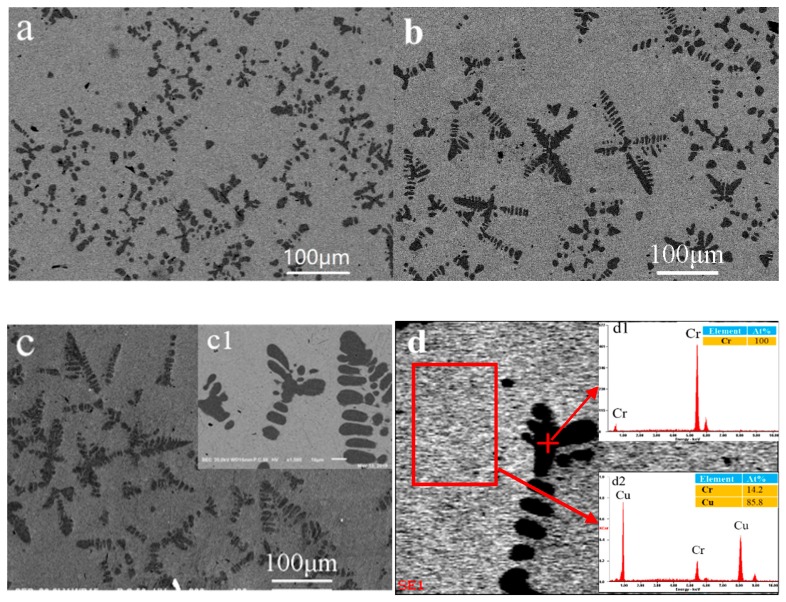
SEM images of as–cast and annealed Cu–14.2%Cr bulk alloy at different temperatures: (**a**) as cast, (**b**) 160 °C, (**c**) 360 °C, (**c1**) of (**c**) (magnification), (**d**) 360 °C, (**d1**) EDS of the particles, and (**d2**) EDS of the Cu–Cr bulk alloy.

Figure 3 shows SEM images of as–cast and annealed Cu–Cr bulk alloy at different temperatures. Unlike the irregular Cr particles obtained in the literature [18], many dendritic particles precipitated on the surface of as–cast Cu–Cr bulk alloy in Figure 3a. According to the XRD patterns in Figure 1a and EDS in Figure 3d1. these particles are pure Cr. It can be revealed that the Cu–Cr alloy prepared by vacuum melting had a very low solid solubility of Cr in Cu. During the cooling, the solid solubility of Cr in Cu drastically decreased; hence, it led to the precipitation of Cr particles. The largest size can reach 30–50 μm. Figure 3b,c show the SEM image of the annealed Cu–Cr bulk alloy at 160 °C, 360 °C, respectively, compared with as–cast Cu–Cr bulk alloy, it was observed that the Cr particles after annealing become more rounded and larger. Relatively regular particles close to a spherical shape can be clearly observed in the Figure 3c,c1. The EDS data in Figure 3d2 show that the content of Cr in Cu–Cr bulk alloy is about 14.2%.

**Figure 4:**
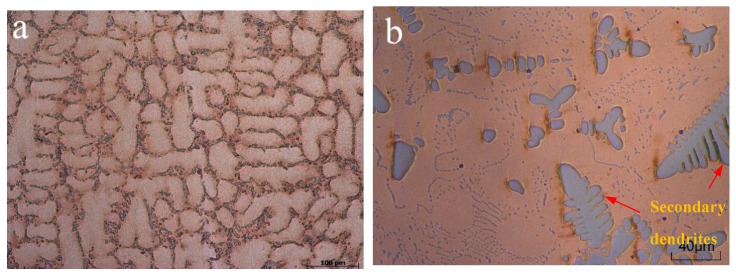
The metallographic of the Cu–Cr bulk alloy: (**a**) as–cast corrosion sample and (**b**) 360 °C annealed sample without corrosion.

Figure 4 shows the metallographic of as–cast and annealed Cu–Cr bulk alloy. Obviously, steel–gray Cr uniformly distributes on the surface of Cu–Cr alloy bulk mainly composed of purple–red Cu in Figure 4a. After annealing, large Cr particles produced secondary dendrites (red arrow) and gradually dispersed, and small Cr particles gradually grew in Figure 4b. Meanwhile, as the amount of Cr particles after annealing increased, and these newly precipitated particles combined with the original particles to cause the particles to grow slightly.

Compared with Figure 2 and Figure 4, the microstructural evolution behavior of Cu–Cr alloy film is obviously different from that of bulk alloy after annealing. Many dendritic Cr particles precipitated on the surface of as–cast and annealed Cu–Cr bulk alloy. Nevertheless, contrast with the smooth surface of as–deposited alloy films, a large number of polyhedral Cu particles (~200 nm) self–formed on the annealed Cu–Cr alloy films’ surface. The fundamental reason for the difference is due to the different preparation methods, which produce a significant difference in the residual stress and atomic diffusion during annealing [3,19]. The Cu–Cr alloy films having high residual stresses and defects were deposited by magnetron sputtering. Li et al. [9] measured the residual tensile stress of magnetron sputtered Cu–Cr thin films on the Si wafers falls in the range of 0.1–0.3 GPa. After annealing, the Cu atoms diffuse along the grain boundary to the film surface, accumulate, nucleate, and grow into small particles in the defects, driven by the residual stress release and energy inside the film. Cu–Cr bulk materials having a very low solid solubility of Cr in Cu are stable materials obtained by vacuum melting. During the cooling and annealing, the solid solubility of Cr in Cu drastically decreased, leading to precipitation and growth of Cr particles and formation of secondary dendrites. Meanwhile, XRD patterns demonstrated that the crystallinity of Cu grains can improve and promote the diffusion of Cr atoms.

### 3.4. SERS Performance of the PI, Cu–Cr Bulk Alloy, and Cu–Cr Alloy Film

**Figure 5:**
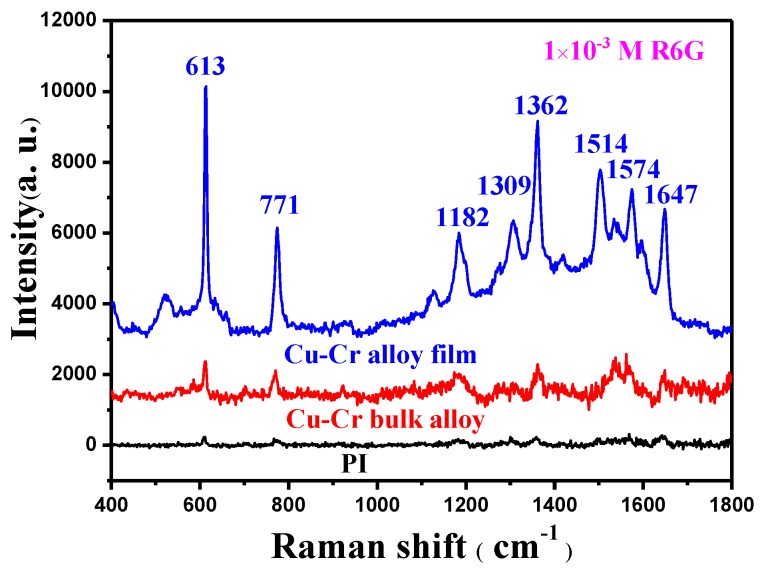
Raman spectra of the PI, 160 °C annealed Cu–14.6%Cr alloy film, and Cu–14.2%Cr bulk alloy.

The PI, 160 °C annealed Cu–14.6%Cr alloy film, and Cu–14.2%Cr bulk alloy were immersed into 1 × 10^–3^ M R6G solution; the Raman spectra is shown in Figure 5. The SERS intensity of the Cu–14.6%Cr alloy film is obviously higher than that of the Cu–14.2%Cr bulk alloy, and the PI has the weak signal. This is because the particle number on the surface of Cu–Cr alloy film in unit area is much larger than that of bulk alloy, which provides quite a lot of hot spots for SERS enhancement, while the PI has a smooth surface with the lowest surface roughness. In addition, the electromagnetic enhancement effect of Cu is far greater than that of Cr [20]. Hence, the SERS enhancement of the Cu particles self–formed on the film surface is stronger than the Cr particles precipitated on the bulk surface. Importantly, in comparing Figure 2b and Figure 3b, it is clear that the gap between the adjacent Cu particles is much narrower than that of the Cr particles. Moreover, in contrast to the smooth surface of the as–deposited Cu–Cr alloy films in Figure 2a, a large number of polyhedral Cu particles (~200 nm) self–formed on the annealed Cu–Cr alloy film’s surface, which can result in nanoscale roughness. However, Cr particles with the size 30–50 μm precipitated on the surface of annealed the Cu–Cr bulk alloy (Figure 3b), leading to micron-scale roughness. According to the literature [21], the SERS substrate with nanoscale surface roughness can adsorb more probe molecules. Previous studies have also found that Ag or Au nanoparticles aggregates with the size of 50–100 nm as SERS substrates possess a very high SERS enhancement. [21,22]. Obviously, the edges of polyhedral Cu particles can produce more hot spots at the tips than those near spherical micro–scale Cr particles [23,24]. In fact, the particle number, particle size, surface roughness, and interstitial gap play important roles in surface enhanced Raman scattering. In the future, with the adjustment of the shape, size, and number of Cu particles, the Cu particles/Cu–Cr alloy film has potential as an effective SERS substrate.

## 4. Conclusions

The microstructural evolution behavior of Cu–Cr alloy films is obviously different from that of bulk alloys after annealing. Many dendritic Cr particles precipitated on the surface of as–cast and annealed Cu–Cr bulk alloy. Nevertheless, compared with the smooth surface of as–deposited alloy films, a large number of polyhedral Cu particles (~200 nm) self–formed on the annealed Cu–Cr alloy film’s surface, which generated larger nanoscale surface roughness. The fundamental reason for the difference is due to the different preparation methods, which produce a significant difference in residual stress and atomic diffusion during annealing. After annealing, the Cu atoms diffuse along the grain boundary to the film surface, accumulate, nucleate, and grow into small particles in the defects, driven by residual stress release and energy inside the film. The larger Cr contents are able to suppress the diffusion of Cu atoms along the grain boundary and surface. On the contrary, the crystallinity of Cu grains in the Cu–Cr bulk alloy improved and promoted the diffusion of Cr atoms. The SERS intensity of the Cu–14.6%Cr alloy film is obviously higher than that of the Cu–14.2%Cr alloy bulk, and the PI has the weak signal, which was attributed to the particle number, surface roughness, and interstitial gap of the SERS substrate. The particle/film composite structure of Cu–Cr alloy film has potential as an effective SERS substrate.
